# Krankenhauseinweisungsgründe für Menschen mit Demenz – ein Scoping-Review

**DOI:** 10.1007/s00391-021-02013-3

**Published:** 2022-04-14

**Authors:** Susanne Stiefler, Ellen Dunker, Annika Schmidt, Anna-Carina Friedrich, Carolin Donath, Karin Wolf-Ostermann

**Affiliations:** 1 Institut für Public Health und Pflegeforschung (IPP), Universität Bremen, Grazer Str. 4, 28359 Bremen, Deutschland; 2 Psychiatrische und Psychotherapeutische Klinik, Friedrich-Alexander-Universität Erlangen-Nürnberg (FAU), Erlangen, Deutschland

**Keywords:** Leichte kognitive Beeinträchtigung, Ambulant sensitive Krankenhausfälle, Krankenhauseinweisung, Literaturübersicht, Mild cognitive impairment, Ambulatory care sensitive conditions, Hospitalization, Literature review

## Abstract

**Hintergrund:**

Krankenhausaufenthalte stellen für Menschen mit Demenz eine hohe Belastung dar, die den Abbau kognitiver und motorischer Fähigkeiten beschleunigen können. Verhaltensänderungen und Orientierungsprobleme können bei Menschen mit Demenz während Krankenhausaufenthalten verstärkt auftreten. Einige Krankenhausaufenthalte sind durch eine bessere ambulante Versorgung potenziell vermeidbar.

**Ziel der Arbeit:**

Erstellung eines aktuellen Überblicks zu den häufigsten Krankenhauseinweisungsgründen für Menschen mit Demenz oder leichten kognitiven Beeinträchtigungen.

**Material und Methoden:**

Zur Erstellung des Scoping-Reviews wurde eine systematische Literaturrecherche in den Datenbanken PubMed®, CINAHL und PsycINFO® im Mai 2020 durchgeführt. Einbezogen wurden Publikationen in deutscher und englischer Sprache, die zwischen Juli 2010 und Mai 2020 publiziert wurden.

**Ergebnisse:**

Die häufigsten Krankenhauseinweisungsgründe, die in den 14 eingeschlossenen Studien genannt wurden, waren Infektionskrankheiten, insbesondere Atemwegs- und Harnwegsinfektionen sowie Herz-Kreislauf-Erkrankungen (allgemein oder spezifisch, z. B. in Form von Herzinsuffizienz), Stürze, Verletzungen, Vergiftungen und Frakturen sowie gastrointestinale Krankheiten.

**Diskussion:**

Bei dem Großteil der häufigsten Krankenhauseinweisungsgründen handelt es sich um potenziell vermeidbare Krankenhausaufenthalte bei rechtzeitiger adäquater ambulanter Versorgung. Eine Stärkung der ambulanten Versorgung von Menschen mit Demenz kann zur Vermeidung von Krankenhausaufenthalten beitragen.

**Zusatzmaterial online:**

Zusätzliche Informationen sind in der Online-Version dieses Artikels (10.1007/s00391-021-02013-3) enthalten.

## Abstract (en)

**Background:**

Hospitalization represents a high burden for people with dementia, which can accelerate the decline of cognitive and motor skills. Behavioral changes and orientation problems may be increased in people with dementia during hospitalization. Some hospitalizations are potentially preventable by improved outpatient care.

**Objective:**

To provide an up to date overview of the most common reasons for hospitalization of people with dementia or mild cognitive impairment.

**Material and methods:**

A systematic literature search was conducted in the databases PubMed®, CINAHL and PsycINFO® in May 2020 to conduct the scoping review. Studies in German and English published between July 2010 and May 2020 were included.

**Results:**

The most common reasons for hospitalization, which were named in the 14 included studies, were infectious diseases, especially respiratory infections and urinary tract infections, cardiovascular diseases (in general or specific, e.g. heart failure) and injuries, poisoning, fractures and falls, and gastrointestinal diseases.

**Conclusion:**

Most of the most common reasons for hospitalization are ambulatory care-sensitive hospital cases. Strengthening outpatient care for people with dementia may help prevent hospitalizations.

## Hintergrund

Derzeit leben in Deutschland ca. 1,6 Mio. Menschen mit Demenz (MmD). Vorausberechnungen prognostizieren einen Anstieg von Demenzerkrankungen auf 2,4–2,8 Mio. bis zum Jahr 2050 [6]. Von einer Einweisung in ein Krankenhaus sind MmD häufiger betroffen als Menschen ohne Demenz (MoD) gleichen Alters [22], das Risiko ist um das 1,4- bis 3,6-Fache erhöht [21]. Dabei ist die Demenzerkrankung oftmals nicht der primäre Einweisungsgrund [25]. In der Literatur finden sich Hinweise darauf, dass es sich bei einigen der Krankenhausaufenthalte um ambulant-sensitive Krankenhausaufenthalte [24] und damit um potenziell vermeidbare Krankenhausaufenthalte, insofern eine rechtzeitige und adäquate ambulante Behandlung stattgefunden hätte, handelt [28]. Vor dem Hintergrund, dass herausfordernde Verhaltensweisen und Orientierungsprobleme in unbekannten Situationen wie Krankenhausaufenthalten verstärkt auftreten, stellen stationäre Aufenthalte für MmD oftmals eine hohe Belastung dar, die den Abbau kognitiver und motorischer Fähigkeiten beschleunigen können [25]. Zudem treten bei MmD häufiger Behandlungskomplikationen auf, und die Mortalität, während oder infolge eines Krankenhausaufenthaltes, ist im Gegensatz zu MoD erhöht [13]. Aus diesem Grund sind nichtnotwendige Krankenhausaufenthalte besonders für MmD und Menschen mit leichten kognitiven Beeinträchtigungen (engl. „mild cognitive impairment“ [MCI]) zu vermeiden.

Die vorliegende Literaturübersicht gibt einen aktuellen Überblick über Krankenhauseinweisungsgründe von MmD oder Menschen mit leichten kognitiven Beeinträchtigungen (MmMCI), aufbauend auf den Literaturübersichten von Toot et al. [25] und Pinkert und Holle [18]. In diesen Publikationen zeigte sich, dass MmD ein erhöhtes Risiko einer Krankenhauseinweisung aufgrund körperlicher gesundheitsbezogener Faktoren, einschließlich orthopädischer, respiratorischer und urologischer Faktoren, im Vergleich zu MoD aufweisen [25]. Ebenfalls wurden Infektionskrankheiten, Frakturen oder ernährungsbedingte Gründe als häufigste Krankenhauseinweisungsgründe berichtet [18]. Mit der vorliegenden Arbeit erfolgte eine Aktualisierung der bis Juni 2010 beschriebenen Ergebnisse.

Eine Aktualisierung der Literaturübersichten ist für die Anpassung der präklinischen medizinischen Versorgung von MmD und MmMCI auf Basis aktueller Daten von Relevanz. Folgende Fragestellung wird untersucht: Was sind die häufigsten Krankenhauseinweisungsgründe für MmD und MmMCI?

Um eine Orientierung über den Stand der Literatur zu dieser Thematik zu geben und die Ergebnisse aus Einzelstudien zu bündeln, wurde die Erstellung eines Scoping-Reviews gewählt. Scoping-Reviews sind geeignet, um das Ausmaß des Wissens zu bewerten und zu verstehen, oder um die Merkmale oder Konzepte in diesem Feld zu identifizieren, zu beschreiben, zu berichten oder zu diskutieren [26].

## Methodisches Vorgehen

Das methodische Vorgehen sowie die Berichtsdarstellung in diesem Artikel orientieren sich an der vom Joanna Briggs Institute (JBI) entwickelten Methodik zur Erstellung von Scoping-Reviews [17].

### Suchstrategie und Auswahlkriterien für Studien

Die Suchstrategie und Auswahlkriterien wurden auf Basis der Arbeiten von Toot et al. [25] und Pinkert und Holle [18] erstellt. Folgende Einschlusskriterien wurden für die vorliegende Arbeit formuliert:

#### Population.

Zielpopulation waren MmD und MmMCI.

#### Konzept.

Einbezogen wurden Publikationen, in denen MmD und MmMCI stationär in einem Krankenhaus aufgenommen wurden und die primäre Ursache für den Krankenhausaufenthalt mittels validen Diagnosekriterien (Diagnostic and Statistical Manual of Mental Disorders (DSM) oder International Classification of Diseases (ICD)), Autopsieberichten oder validen Assessments (z. B. Mini-Mental State Examination (MMSE)) ggf. mit Vergleichsgruppen (z. B. MoD) analysiert wurde.

#### Kontext.

Um den aktuellen Forschungsstand (nach Toot et al. [25]) abzubilden, wurden Arbeiten in deutscher oder englischer Sprache mit Publikationsdatum ab Juli 2010 berücksichtigt.

#### Evidenzquellen.

Beobachtungsstudien mit quantitativen Analysen, systematische Übersichtsarbeiten und Metaanalysen wurden in die Ergebnissynthese einbezogen.

Ausgeschlossen wurden Studien, die

- Eintrittsgründe in die stationäre Langzeitpflege, stationäre Palliativversorgung, teilstationäre und tagesklinische Versorgung beschreiben,
- spezifische Erkrankungen oder Einweisungsgründe untersuchen oder
- nicht frei verfügbar waren.

Die Suche wurde in den Datenbanken CINAHL, PubMed® und APA PsycInfo® am 20.05.2020 durchgeführt. Eine detaillierte Beschreibung der Suchstrategie, des Screenings und Auswahlverfahrens sowie der Datenextraktion sind dem Zusatzmaterial online: Supplement 1 zu entnehmen.

### Definitionen

Die Darstellung der Krankenhauseinweisungsgründe basiert auf in den Publikationen berichteten Diagnosen, teils zusammengefasst in Diagnosegruppen. Als Gründe bezeichnete Krankenhausaufnahmen, die augenscheinlich auf Diagnosen beruhen, wurden ebenfalls berücksichtigt. Ungenau beschriebene Einweisungsgründe wie „weitere Erkrankungen“ werden nicht berichtet.

### Ergebnissynthese und -darstellung

Um eine Darstellung von Einzelfällen auszuschließen und verallgemeinerbare Aussagen zu treffen, werden nur Krankenhauseinweisungsgründe dargestellt, die in den Einzelstudien mit einer Häufigkeit von mindestens 8 % vorkamen. Unterhalb der 8 %-Grenze sind vermehrt Einzeldiagnosen und -gründe beschrieben, weshalb von einer Darstellung dieser abgesehen wird. Von Studien, die sowohl MmD als auch MmMCI untersuchen, werden nur Ergebnisse für MmD berichtet. Ergebnisse für MmMCI werden im Zusatzmaterial online: Supplement 3, Tabelle S3a berichtet. Die Gründe werden in übergeordnet passende Kategorien zusammengeführt dargestellt und in Klammern die Spannweite der Häufigkeitsdarstellung berichtet.

## Ergebnisse

**Figure 1:**
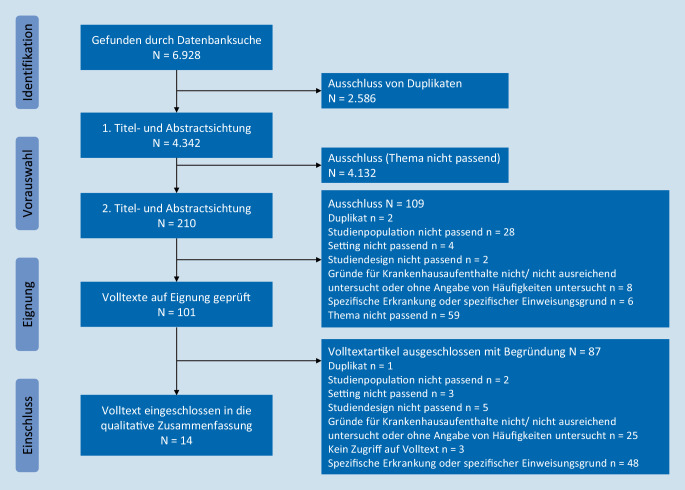

In die Ergebnisdarstellung wurden 14 Arbeiten (3 prospektive Kohortenstudien [8, 14, 20], 4 retrospektive Kohortenstudien [1, 5, 10, 23] und 7 Querschnittsstudien [2, 3, 7, 9, 15, 16, 29]) einbezogen. Den Trefferverlauf und den Ein- und Ausschluss von Studien zeigt Abb. 1.

Im Zusatzmaterial online: Supplement 2 sind Basisinformationen der Einzelstudien zusammengefasst. Die Studienpopulationen umfassten zwischen 70 und 127.227 Personen [1, 14]; der Median liegt bei 879 Personen, für jene Studien, die Angaben zur Studienpopulation machten. Sieben Studien führten einen Vergleich von MmD/MmMCI und MoD durch [1, 3, 5, 7, 9, 15, 29]. Das arithmetische Mittel des berichteten Alters der Studienteilnehmenden liegt zwischen 75,8 und 86,2 Jahren für MmD [10, 20] und zwischen 80,7 und 86 Jahren für MmMCI [3, 7].

### Gründe für Krankenhauseinweisungen

In 11 übergeordneten Kategorien lassen sich 40 Einweisungsgründe mit Häufigkeiten ≥ 8 % berichten; 68 Einweisungsgründe, die mit einer Häufigkeit < 8 % beschrieben werden (Zusatzmaterial online: Supplement 3, Tabelle S3b), lassen sich in 19 Kategorien zusammenfassen. Die geringste berichtete Häufigkeit liegt bei 0,03 % (Erkrankungen der weiblichen Geschlechtsorgane) [7], die höchste bei 41 % (Pneumonie) [8]. Demenz wird als Einweisungsgrund mit einer Häufigkeit < 8 % beschrieben [10, 16]. In den meisten Studien (*n* = 10) wurden Infektionskrankheiten als Krankenhauseinweisungsgründe angegeben. Dabei beschrieben 2 Studien Infektionskrankheiten (19–20,6 %) allgemein bzw. andere Infektionserkrankung als eine Pneumonie als Einweisungsgrund [1, 8]. In 8 Studien wurden dabei Atemwegsinfektionen (8,7–41 %) [1, 8] als Einweisungsgrund dargestellt, teilweise konkretisiert als Pneumonie [2, 8, 10, 15] und in einer Studie zusammengefasst mit anderen Infektionskrankheiten [14]. Zwei Studien berichteten Harnwegsinfektionen (8,6–11 %) [2, 23] als Einweisungsgrund. In 5 Studien werden Herz-Kreislauf-Erkrankungen (12,9–22,5 %) [3, 9] berichtet, entweder allgemein als Herz-Kreislauf-Erkrankungen bezeichnet oder auch spezifischer als Herzinsuffizienz und ischämische Herzerkrankung. Ebenfalls in jeweils 5 Arbeiten wurden gastrointestinale Erkrankungen (8–12,9 %) [3, 8] oder Verletzungen, Vergiftungen, Frakturen oder Stürze (11,7–26 %) [1, 20] als Einweisungsgründe aufgeführt. Vier Studien berichteten Bewusstseinsstörungen und psychologische Symptome (9,4–40 %) [7, 23], je 2 Studien Nieren- und urologische Erkrankungen (11,9–27,3 %) [7, 10] bzw. muskuloskeletale Erkrankungen (9,8–14,5 %) [3, 14]. In je einer Studie wurden rheumatische Erkrankungen (8,9 %) [7] oder ernährungsbedingte Gründe genannt 22 % [15]. Die häufigsten Krankenhauseinweisungsgründe fasst Tab. 1 zusammen. Für MmMCI zeichnen sich größtenteils die gleichen Krankenhauseinweisungsgründe ab.

**Table 1:** 

Kategorie Krankenhauseinweisungsgrund^a^	Krankenhauseinweisungsgrund, Studienergebnis	Häufigkeit (in %)
Infektionskrankheiten: unspezifisch	Infektionen	20,6 [1]
	Andere Infektionen als Pneumonie	19 [8]
Infektionskrankheiten: Atemwegsinfektionen	Atemwegserkrankungen	22,4 [9] 18,7 [7] 8,7 [1]
	Pneumonie	41 [8] 20,5 [2] 16 [15] 10,1 [10]
	Atemwegsinfektionen und Infektionskrankheiten	31,4 [14]
Infektionskrankheiten: Harnwegsinfektion	Harnwegsinfektion	11 [23] 8,6 [2]
Krankheiten des Herz-Kreislauf-Systems	Krankheiten des Kreislaufsystems	22,5 [3]^b^ 12,9 [9]
	Chronische ischämische Herzerkrankung	17 [20]
	Kardiovaskuläre Erkrankungen	16,6 [1] 15,7 [14]
	Herzinsuffizienz, kardiovaskuläre Erkrankungen	13,6 [7]
Gastrointestinale Krankheiten	Krankheiten des Verdauungssystems	12,9 [3]
	Gastrointestinale Probleme	11,8 [14]
	Gastrointestinale Erkrankung	9 [20]
	Krankheiten des digestiven Systems	8,6 [1]
	Gastrointestinale Blutungen	8 [8]
Stürze, Verletzungen, Vergiftungen, Frakturen	Synkope, Sturz, Trauma^c^	26 [20]
	Stürze	24 [23]
	Verletzungen, Vergiftungen und bestimmte andere Folgen äußerer Ursachen	19,9 [3]
	Stürze, Frakturen, Osteoporose	12,4 [7]
	Verletzungen und Vergiftungen	11,7 [1]
Bewusstseinsstörungen und psychologische Symptome	Halluzinationen oder Verwirrtheit	40 [23]
	Verhaltensbezogene psychische Demenzsymptome (BPSD)	23 [15]
	Delirium	14,5 [16]
	Neuropsychiatrische Erkrankungen	16,6 [9] 9,4 [7]
Nieren- und urologische Erkrankungen	Chronisches Nierenversagen	27,3 [10]
	Andere Erkrankungen des Harnsystems	20,5 [10]
	Nieren- und urologische Erkrankungen	11,9 [7]
Muskuloskeletale Erkrankungen	Krankheiten des Muskel-Skelett-Systems und des Bindegewebes	14,5 [3]
	Orthopädische Erkrankungen	9,8 [14]
Rheumatische Erkrankungen	Rheumatische Erkrankungen	8,9 [7]
Ernährungsbedingte Gründe	Probleme bei der Nahrungsaufnahme	22 [15]

^a^Zuliani et al. und Daiello et al. berichten keinen Krankenhauseinweisungsgrund ≥ 8 %

^b^Bickel et al. berichten mehrere Krankenhauseinweisungsgründe pro Person, Summe > 100 %

^c^Datenlage lässt keine Erklärung zu, ob Synkope als Folge eines Sturzes gemeint ist

## Diskussion

Dieses Scoping-Review liefert eine aktuelle umfassende Darstellung von Krankenhauseinweisungsgründen und beantwortet dadurch zusammenfassend die Forschungsfrage, welches die häufigsten Krankenhauseinweisungsgründe für MmD und MmMCI sind.

Die einbezogenen Studien betrachteten MmD als Zielgruppe sowie vereinzelt auch MmMCI [3, 7]. Die häufigsten Krankenhauseinweisungsgründe für diese Zielgruppe sind Infektionskrankheiten, gefolgt von Erkrankungen des Herz-Kreislauf-Systems, Stürzen, Verletzungen, Vergiftungen und Frakturen sowie gastrointestinalen Erkrankungen. Zudem finden sich jedoch auch Gründe für Krankenhausaufenthalte, die lediglich in jeweils einer Studie angeführt sind, wie Probleme bei der Nahrungsaufnahme [15]. Die Studienergebnisse sind im Vergleich zur der Recherche zugrunde liegenden Literatur [18, 25] weitestgehend gleichbleibend. Dies unterstreicht den Bedarf an spezifischen Interventionen, die an vermeidbaren Krankenhauseinweisungen für MmD und MmMCI ansetzen. Im Rahmen einer randomisierten kontrollierten Studie wird derzeit die Wirksamkeit einer solchen spezifischen Intervention untersucht [12].

Durch ein nicht-pharmakologisches Management lassen sich spezifische Einweisungsgründe adressieren, die mit einer hohen Häufigkeit berichtet wurden, wie z. B. Stürze mit bis zu 26 % [20] oder verhaltensbezogene psychische Demenzsymptome mit 23 % [15]. Auf Letztere ist ein besonderer Fokus zu legen, da sich herausfordernde Verhaltensweisen u. a. durch die psychische Belastung im Rahmen eines Krankenhausaufenthalts beispielsweise durch die unbekannte Umgebung verstärken können [21]. Durch nicht-pharmakologische Interventionen, die an demenzspezifischen Einweisungsgründen wie Stürze, Bewusstseinsstörungen und psychologische Symptome ansetzen, ließen sich Krankenhausaufenthalte ggf. vermeiden.

Sowohl Herz-Kreislauf-Erkrankungen als auch respiratorische Erkrankungen gelten als häufigste ambulant-sensitive Erkrankungen [24]. Nach Sundmacher et al. [24] sind voraussichtlich über 60 % der Krankenhausaufnahmen aufgrund einer chronischen ischämischen Herzkrankheit, Herzinsuffizienz, weiterer Herz-Kreislauf-Erkrankungen (u. a. Herzklappenerkrankungen, nicht näher bezeichnete Arrhythmien, Arteriosklerose) und Bronchitis/chronisch obstruktive Lungenerkrankung (COPD) vermeidbar und könnten durch die Verbesserung einer kontinuierlichen ambulanten Versorgung reduziert werden. Wolf et al. [28] untersuchten spezifisch für MmMCI und MmD die Häufigkeit von Krankenhausaufnahmen aufgrund ambulant-sensitiver Erkrankungen und kamen zu dem Ergebnis, dass die häufigsten Krankenhausaufnahmen aufgrund von Herz-Kreislauf-Erkrankungen und Erkrankungen des digestiven Systems sowie aufgrund gutartiger oder bösartiger Neubildungen erfolgten. Es wird davon ausgegangen, dass etwa jeder 4. Krankenhausaufenthalt von MmD und MmMCI vermeidbar ist [12, 28]. Diese Gegebenheit unterstreicht die Relevanz, die Gründe hierfür zu untersuchen und zu kennen [28]. Zudem scheinen Infektionserkrankungen spezifisch für MmD und MmMCI von besonderer Bedeutung zu sein, da sie im Vergleich zur allgemeinen Bevölkerung in Deutschland eine untergeordnete Rolle als Krankenhauseinweisungsgrund spielen [11]. Geeignete Maßnahmen zur Reduktion ungeplanter Krankenhausaufenthalte können Schulungen und Verbesserung des Selbstmanagements, Bewegung und Rehabilitation sowie der Einsatz von Telemedizin bei ausgewählten Patientengruppen sein [19].

Limitationen dieser Arbeit sind in der Uneinheitlichkeit der Diagnosekriterien der verschiedenen Studien zu sehen. Dies bezieht sich sowohl auf die Demenzdiagnose oder Einstufung von MmMCI als auch auf die Erfassung der Krankenhauseinweisungsgründe. Mitunter sind Diagnosen nicht ausreichend beschrieben, als dass sich für spezifische Diagnosen ein Vergleich der Studien ziehen ließe. Teilweise werden konkrete ICD-Codes der Einweisungs- oder Entlassungsdiagnosen nicht genannt. Somit ist der Abstraktionsgrad der untersuchten Einweisungsgründe unterschiedlich. Um dieser Gegebenheit Rechnung zu tragen, wurden in diesem Scoping-Review die Krankenhauseinweisungsgründe in übergeordnete Kategorien zusammengefasst. Zu berücksichtigen ist weiterhin, dass die Ergebnisse der Studien, die nicht in Deutschland durchgeführt wurden, auf das deutsche Gesundheitssystem möglicherweise nur bedingt übertragbar sind. Grundsätzlich sind die Ergebnisse trotz unterschiedlicher Herkunftsländer weitestgehend homogen. Daher ist davon auszugehen, dass die häufigsten Krankenhauseinweisungsgründe für MmD in dieser Arbeit realitätsnah zusammengefasst wurden. Weitere Limitationen ergeben sich durch unterschiedliche methodische Ansätze in den Studien mit eigenen Stärken und Schwächen. So wurde keine einheitliche Datenbasis verwendet. Die Ergebnisse beruhen teilweise auf Daten aus Patientenakten oder Versicherungsdaten, teilweise auf direkten Assessments mittels Interviews. Der Umfang der Studienpopulationen unterschied sich zudem deutlich. Häufigkeitsdarstellungen der Krankenhauseinweisungsgründe für kleinere Studienpopulationen waren meist deutlich höher [8, 14, 15], weshalb ein Vergleich der Häufigkeiten sich nicht als sinnvoll erwies.

## Implikationen für die Praxis

Die Arbeit bündelt aktuelle Krankenhausaufnahmegründe für MmD und MmMCI. Viele davon werden in der Literatur als vermeidbare Krankenhauseinweisungsgründe genannt, insofern eine rechtzeitige und adäquate Behandlung stattgefunden hätte. Daher sollte die ambulante Versorgung für MmD oder MmMCI gezielter auf die Vermeidung von stationären Aufenthalten bei Infektionserkrankungen, Herz-Kreislauf-Erkrankungen und ernährungsbedingten Problemen ausgelegt werden. Hierfür sind die beteiligten Akteure in der gesundheitlichen Versorgung wie Hausärzte und Pflegekräfte zu sensibilisieren. Ein verstärkter Einsatz von Risikoassessments und Screenings in pflegerischen und/oder häuslichen Settings könnte sinnvoll sein, im Sinne eines frühen Erkennens von vermeidbaren Krankenhauseinweisungen. Aber auch eine umfassende Sensibilisierung der Angehörigen sowie Betreuerinnen und Betreuer von MmD und MmMCI kann dazu beitragen, Krankenhauseinweisungen zu reduzieren, etwa wenn die jeweiligen Bezugspersonen bei einer optimalen Ausschöpfung und Durchführung der ambulanten Versorgung unterstützen. Bestehende Leitlinien wie nationale Versorgungsleitlinien leisten bereits einen wichtigen Beitrag zur Optimierung der ambulanten Versorgung. Weiterhin ist die Stärkung der kontinuierlichen Versorgung auf struktureller Ebene [24] möglich und sinnvoll – beispielsweise durch eine gute Koordination der ambulanten Leistungserbringer [28]. Hierbei könnte wie in der nationalen Demenzstrategie gefordert, der von der Bundesärztekammer empfohlenen Einsatz von Demenzbeauftragten in den Landesärztekammern einen Beitrag leisten, um den Kammerangehörigen in der Versorgung von MmD und Fortbildungsmöglichkeiten beratend zur Seite zu stehen [4]. Effektives Management chronischer Erkrankungen (etwa mittels Disease-Management-Programmen) [24] sowie eine effektive Behandlung akuter Krankheiten, das frühe Erkennen von Erkrankungen und primärpräventive Maßnahmen spielen ebenso eine zentrale Rolle [24]. Eine adäquate Behandlung von nichtkognitiven Symptomen bei MmD und MmMCI durch möglichst nicht-pharmakologische Maßnahmen kann zudem dazu beitragen, dadurch bedingte Krankenhausaufenthalte zu vermeiden. Vor allem im Hinblick auf Bewusstseinsstörungen und psychologische Symptome als Einweisungsgrund könnte weiterhin die stationsäquivalente psychiatrische Behandlung nach § 115d SGB V für MmD und MmMCI geeignet sein [27]. Zum Lebensende hin kann mit Advance Care Planning, unter Berücksichtigung des Willens der MmD und MmMCI bei der Organisation ihrer palliativen Versorgung, eine ambulante Betreuungssituation entstehen, die nichtnotwendige und ungewollte Krankenhauseinweisungen reduzieren kann.

## Supplementary Information






