# Supplementary material for: Reasons for hospitalization of people with dementia—A scoping review
Source: Z Gerontol Geriatr. 2022 Apr 14;56(1):42–7. [Article in German] doi: 10.1007/s00391-021-02013-3 (PMC9876850; doi:10.1007/s00391-021-02013-3)
Supplement: Supplementary file 1 [file 391_2021_2013_MOESM1_ESM.docx]

# Supplement 1

## Recherchestrategie

In Tabelle S1 sind die Suchbegriffe zusammengefasst. Die Oberkategorien für Erkrankungen des demenziellen Formenkreises und MCI sowie für stationäre Krankenhausaufenthalte wurden mit dem Booleschen Operator „AND“ verknüpft und durch die weiteren Suchbegriffe mit „OR“ verbunden.

Tabelle S1: Suchbegriffe und ihre Verknüpfungen

| Oberbegriff | Suchbegriffe (Titel und Abstract) | Suchbegriffe (Medical Subject Headings bzw. Exact Subject Headings) |
| --- | --- | --- |
| demenzielle Erkrankung oder leichte kognitive Beeinträchtigungen | dementia  alzheimer*  "mild cognitive impairment"  "cognitive impairment"  "cognitive decline"  "cognitive loss"  "cognition loss"  "cognitive dysfunction" | PubMed/ Medical Subject Headings:  dementia  CINAHL/ Exact Subject Headings:  "Dementia"  "Frontotemporal Dementia"  "Delirium, Dementia, Amnestic, Cognitive Disorders"  "Dementia, Multi-Infarct”  "AIDS Dementia Complex"  "Lewy Body Disease"  "Dementia, Senile"  "Dementia, Presenile"  "Dementia Patients"  PsycINFO/ Exact Subject Headings:  dementia |
| stationärer Krankenhausaufenthalt | hospitalization  hospitalisation  "hospital admission"  "inpatient admission"  "stationary admission"  "hospital admittance” | PubMed/ Medical Subject Headings:  hospitalization  CINAHL/ Exact Subject Headings:  "Hospitalization"  "Inpatients"  "Hospitals, Public"  PsycINFO/ Exact Subject Headings:  hospitalization |

## Screening und Auswahlverfahren

Die Sichtung der Treffer wurde mit den Softwares Rayyan QCRI [16] und Covidence [10] durchgeführt. Nach dem Entfernen von Duplikaten erfolgte eine erste Selektion der Artikel anhand der Titel und Abstracts durch zwei unabhängige Personen (ED und SSt), um Arbeiten auszuschließen, die eindeutig andere Themen adressierten. Im zweiten Schritt wurde ein differenzierteres Titel- und Abstractscreening sowie das anschließende Volltextscreening durch dieselben Personen durchgeführt. Bei Uneinigkeit über den Einbezug einer Publikation wurde eine dritte Person herangezogen (AS). Dieses Vorgehen wurde gewählt, um eine hohe Interraterreliabilität zu gewährleisten. Im Anschluss wurden die Referenzen der einbezogenen Publikationen auf weitere relevante Arbeiten geprüft (ED).

## Datenextraktion

Die Datenextraktion erfolgte mit der Software Covidence [10] durch eine Person (ED). Zur Gewährleistung einer hohen Validität, wurden die tabellarisch erfassten Ergebnisse der Datenextraktion durch eine weitere Person für alle dargestellten Ergebnisse überprüft (SSt). Extrahiert wurden die Namen der Autorinnen und Autoren, das Publikationsdatum, das Herkunftsland der Studie, das Studiendesign, die Datenbasis und Messinstrumente, die Beschreibung der Population (ggf. der Vergleichsgruppen), die Form der Demenzerkrankung bzw. kognitiven Beeinträchtigung und die Gründe sowie die Häufigkeit der genannten Gründe für Krankenhausaufenthalte.
