# Supplementary material for: Reasons for hospitalization of people with dementia—A scoping review
Source: Z Gerontol Geriatr. 2022 Apr 14;56(1):42–7. [Article in German] doi: 10.1007/s00391-021-02013-3 (PMC9876850; doi:10.1007/s00391-021-02013-3)
Supplement: Supplementary file 2 [file 391_2021_2013_MOESM2_ESM.docx]

# Supplement 2

Tabelle S2: Allgemeine Studieninformationen der einbezogenen Studien

| **Autorenschaft (Publikationsjahr)** | **Herkunft** | **Studienpopulation**  **Stichprobengröße** | **Spannweite der  Häufigkeiten von Krankenhauseinweisungs-gründen** | **Studiendesign/ Datengrundlage für Krankenhausaufenthalte** | **Messinstrument Demenz/ MCI** | **Vergleichsgruppe** |
| --- | --- | --- | --- | --- | --- | --- |
| Allers & Hoffmann (2018) [1] | DEU | N = 127.227  (n = 58.713 MmD  n = 68.514 MoD) | 1,0 %-20,6 % | Retrospektive Kohortenstudie: Krankenkassendaten | ICD-10 | MmD und MoD |
| Bernardes et al. (2018) [2] | PRT | N = 288.096 Krankenhausaufnahmen von MmD | 2,2 %-20,5 % | Querschnittstudie:  nationale Krankenhausdatenbank portugiesischer öffentlicher Krankenhäuser | ICD-9 | -- |
| Bickel et al. (2018) [3] | DEU | N = 1.469^[[1]](#footnote-1)^  (n = 270 MmD  n = 290 MmMCI  n = 881 MoD) | 2,1 %-22,5 % | Querschnittstudie:  standardisierte Interviews mit verantwortlicher Pflegekraft, Patientenakte | DSM-4  6-CIT | MmD und MoD  MmMCI und MoD |
| Daiello et al. (2014) [4] | USA | N = 25.839  (n = 3.908 MmD  n = 21.931 MoD)  Krankenhausaufnahmen | 5,1 %-7,0 % | Retrospektive Kohortenstudie: administrative Medicare-Leistungsdaten | ICD-9  demenz-spezifische Medikamenten-verschreibung | MmD und MoD |
| Fogg et al. (2018) [5] | GBR | N = 13.652  (n = 19,8 % MmD  n = 11,6 % MmMCI  n = 68,9 % MoD) ^[[2]](#footnote-2)^  N = 19.269 Krankenhausaufnahmen | 0,03 %-18,7 % | Querschnittstudie:  Patientenakte | AMTS | MmD und MoD  MmMCI und MoD |
| Givens et al. (2012) [7] | USA | N = 323 MmD | 3,0 %-41,0 % | Prospektive Kohortenstudie: Pflegeheimbewohnerinnen und Pflegeheimbewohner-Assessments und Telefoninterviews | CPS  MMSE  GDS | -- |
| Guijarro et al. (2010) [8] | ESP | N = 3.354.071  (n = 40.482 MmD  n = 3.313.589 MoD)  Krankenhausaufnahmen | 12,9 %-22,4 % | Querschnittstudie:  Routinedaten des andalusischen Gesundheitsdienstes | ICD-9 | MmD und MoD |
| Gungabissoon et al. (2020) [9] | GBR | N = 5.218 MmD | 2,3 %-27,3 % | Retrospektive Kohortenstudie:  elektronische Patientenakte | ICD-10  MMSE  HoNoS | -- |
| Lin et al. (2017) [14] | TWN | N = 70 MmD | 7,8 %-31,4 % | Prospektive Kohortenstudie: Gesundheitsdaten des Pflegeheims für erstmaligen Krankenhausaufenthalt nach Einzug ins Pflegeheim | DSM-4  NINCDS-ADRDA Alzheimer Kriterien | -- |
| Matsuoka et al. (2019) [15] | JPN | N = 128  (n = 100 MmD  n = 28 MoD) | 4,00 %-23,0 % | Querschnittstudie:  Patientenakte | Autopsieberichte | MmD und MoD |
| Patira et al. (2017) [17] | USA | N = 131 MmD | 4,5 %-14,5 % | Querschnittstudie:  elektronische Patientenakte | ICD-9  Validierung durch Arzt | -- |
| Rudolph et al. (2010) [20] | USA | N = 879 MmD | 5,00 %-26,0 % | Prospektive Kohortenstudie:  Krankenversicherungsdaten (Medicare Provider Analysis and Review (MedPAR)) | NINCDS-ADRDA Alzheimer Kriterien | -- |
| Spears et al. (2019) [23] | USA | N = 879 MmD | 1,0 %-40,0 % | Retrospektive Kohortenstudie: elektronische Patientenakte | ICD-9  ICD-10 | -- |
| Zuliani et al. (2012) [28] | ITA | N = 51.838  (n = 4.466 MmD  n = 47.372 MoD) | 2,8 %-6,8 % | Querschnittstudie:  Entlassungsakten | ICD-9 | MmD und MoD |
| **Legende:** DEU = Deutschland, PRT = Portugal, TWN = Taiwan, USA = Vereinigte Staaten von Amerika, GBR = Vereinigtes Königreich, JPN = Japan, ITA = Italien, MmD = Menschen mit Demenz, MoD = Menschen ohne Demenz, MmMCI = Menschen mit leichten kognitiven Beeinträchtigungen, ICD = International Classification of Diseases, DSM = Diagnostic and Statistical Manual of Mental Disorders, 6CIT = Six-item Cognitive Impairment Test, AMTS = Abbreviated Mental Test Score, CPS = Cognitive Performance Score, MMSE = Mini Mental State Examination, GDS = Global Deterioration Scale, HoNoS = Health of the Nation Outcome Scales, NINCDS-ADRDA Alzheimer Kriterien = Kriterien des [National Institute of Neurological and Communicative Disorders and Stroke](https://en.wikipedia.org/wiki/National_Institute_of_Neurological_and_Communicative_Disorders_and_Stroke) und der [Alzheimer's Disease and Related Disorders Association](https://en.wikipedia.org/wiki/Alzheimer%27s_Disease_and_Related_Disorders_Association) | | | | | | |

1. n = 1 fehlend aufgrund unvollständiger Daten zur Diagnostik [↑](#footnote-ref-1)
2. ausschließlich Angaben zur prozentualen Verteilung/ kein n berichtet [↑](#footnote-ref-2)
