# Supplementary material for: Reasons for hospitalization of people with dementia—A scoping review
Source: Z Gerontol Geriatr. 2022 Apr 14;56(1):42–7. [Article in German] doi: 10.1007/s00391-021-02013-3 (PMC9876850; doi:10.1007/s00391-021-02013-3)
Supplement: Supplementary file 3 [file 391_2021_2013_MOESM3_ESM.docx]

# Supplement 3

Tabelle S3a: In den Studien berichtete Krankenhauseinweisungsgründe für Menschen mit leichten kognitiven Einschränkungen

| **Kategorie Krankenhauseinweisungsgrund** | **Krankenhauseinweisungsgrund, Studienergebnis** | **Häufigkeit** |
| --- | --- | --- |
| Infektionskrankheiten: unspezifisch | Infektionskrankheiten | 5,3 % [5] |
|  | Infektiöse Erkrankungen | 4,4 % [3]^[[1]](#footnote-1)^ |
| Infektionskrankheiten: Atemwegsinfektionen | Atemwegserkrankungen | 16,1 % [5] |
|  | Erkrankungen des Atmungssystems | 9,2 % [3] |
| Krankheiten des Herz-Kreislaufsystems | Krankheiten des Kreislaufsystems | 22,5 % [3] |
|  | Herzinsuffizienz, kardiovaskuläre Erkrankungen | 13,0 % [5] |
| Gastrointestinale Krankheiten | Krankheiten des Verdauungssystems | 12,9  % [3] |
|  | gastrointestinale Erkrankung | 4,2 % [5] |
| Stürze, Verletzungen, Vergiftungen, Frakturen | Verletzungen, Vergiftungen | 19,9 % [3] |
|  | Stürze, Frakturen, Osteoporose | 10,5 % [5] |
|  | Trauma | 3,6 % [5] |
| Bewusstseinsstörungen und psychologische Symptome | Neuropsychiatrische Erkrankungen | 10,6 % [5] |
| Nieren- und urologische Erkrankungen | Nieren- und urologische Erkrankungen | 13,5 % [5] |
|  | Erkrankungen des Urogenitalsystems | 7,7 % [3] |
| Muskuloskelettale Erkrankungen | Muskel- und Skeletterkrankungen | 14,5 % [3] |
| Rheumatische Erkrankungen | rheumatische Erkrankungen | 9,6 % [5] |
| Neubildungen | Neubildungen | 9,9 % [3] |
|  | Krebserkrankung | 2,3 % [5] |
| Endokrine und Blutkrankheiten | Endokrine Erkrankungen | 7,0 % [3] |
|  | Krankheiten des Blutes | 2,6 % [3] |
| Nervenkrankheiten | Erkrankungen des Nervensystems | 2,1 % [3] |
| Hauterkrankungen | Erkrankungen der Haut | 2,1 % [3] |
|  | Hautkrankheiten | 1,9 % [5] |
| Hals-Nasen-Ohren- und Augenerkrankungen | Hals-Nasen-Ohren- und Augenerkrankungen | 1,1 % [5] |

Tabelle S3b: In den Studien berichtete Krankenhauseinweisungsgründe mit einer Häufigkeit < 8 %

| **Kategorie Krankenhauseinweisungsgrund** | **Krankenhauseinweisungsgrund, Studienergebnis** | **Häufigkeit** |
| --- | --- | --- |
| Infektionskrankheiten: unspezifisch | Infektiöse Erkrankungen | 4,4 % [3] |
|  | Infektionskrankheiten | 3,9 % [5] |
| Infektionskrankheiten: Atemwegsinfektionen | Pneumonie | 7,0 % [12]  6,0 % [11]  5,1 % [4] |
|  | Akute Bronchitis | 5,1 % [2] |
|  | Pneumonie/ Rippenfellentzündung mit Komplikationen | 4,0 % [13] |
|  | unspezifische akute Infektionen der unteren Atemwege | 4,0 % [7] |
|  | Chronisch obstruktive Lungenerkrankung | 3,4 % [7] |
|  | Aspirationspneumonitis | 2,2 % [2] |
| Infektionskrankheiten: Harnwegsinfektion | Harnwegsinfektionen | 7,0 % [4] |
| Krankheiten des Herz-Kreislaufsystems | Zerebrovaskuläre Erkrankungen | 7,1 % [1] |
|  | Hirninfarkt | 3,9 % [7] |
|  | zerebrovaskuläre Erkrankung/ keine transitorische ischämische Attacke | 6,8 % [13] |
|  | Akute zerebrovaskuläre Erkrankungen | 6,3 % [2] |
|  | Herzerkrankung | 6,0 % [12] |
|  | Herzerkrankung/ Schock | 5,7 % [13] |
|  | Herzinsuffizienz | 5,7 % [4]  3,0 % [6]  2,3 % [7] |
|  | transitorische ischämische Attacke | 3,1 % [13] |
|  | zerebrovaskuläre Erkrankung mit Komplikationen | 2,8 % [13] |
|  | intrakranielle Blutung | 2,0 % [12] |
|  | Schlaganfall | 1,0 % [12] |
| Gastrointestinale Krankheiten | gastrointestinale Erkrankung | 7,0 % [12]  5,2 % [5] |
| Stürze, Verletzungen, Vergiftungen, Frakturen | Fraktur | 6,0 % [9]  5,0 % [6] |
|  | Oberschenkelhalsfraktur | 6,0 % [7]  3,2 % [2] |
|  | Trauma | 3,5 % [5] |
|  | Offene Wunde am Kopf | 3,0 % [7] |
|  | Oberflächliche Verletzung des Kopfes | 2,8 % [7] |
| Bewusstseinsstörungen und psychologische Symptome | Delirium, Demenz und andere kognitive Störungen | 6,5 % [2] |
|  | Senilität | 6,0 % [7] |
|  | Synkope und Kollaps | 5,1 % [7] |
|  | Delirium oder Veränderung des mentalen Status | 5,0 % [11] |
|  | Sonstige Symptome, die das Erkennungsvermögen und das Bewusstsein betreffen | 3,7 % [7] |
|  | Psychiatrische Erkrankungen | 3,6  % [1] |
|  | Demenz | 4,5 % [10] |
|  | Nicht näher bezeichnete Demenz | 3,0 % [7] |
|  | Synkope | 1,0 % [12] |
|  | Erkrankungen des Urogenitalsystems | 7,7 % [3] |
|  | Urogenitale Störungen | 3,0 % [12] |
|  | Akutes Nierenversagen | 2,3 % [7] |
| Muskuloskelettale Erkrankungen | Erkrankungen des muskuloskelettalen Systems | 1,0 % [1] |
| Ernährungsbedingte Gründe | Dysphagie | 7,0 % [9] |
|  | Flüssigkeits- und Elektrolytstörungen | 2,6 % [2] |
| Neubildungen | Krebserkrankung | 4,3 % [1]  1,2 % [5] |
| Endokrine und Blutkrankheiten | Endokrine Erkrankungen | 7,0 % [3] |
|  | Sepsis | 6,0 % [12] |
|  | Krankheiten des Blutes | 2,6 % [3] |
|  | Septikämie | 2,6 % [2] |
|  | Diabetes mellitus mit Komplikationen | 2,2 % [2] |
| Nervenkrankheiten | Neurologische Erkrankungen | 7,8 % [8] |
|  | Erkrankungen des Nerven- und des sensorischen Systems | 3,2 % [1] |
|  | Erkrankungen des Nervensystems | 2,1 % [3] |
| Hauterkrankungen | Erkrankungen der Haut | 2,1 % [3] |
|  | Hautkrankheiten | 1,8 % [5] |
| Hals-Nasen-Ohren- und Augenerkrankungen | Hals-Nasen-Ohren- und Augenerkrankungen | 0,7 % [5] |
| Erkrankungen der weiblichen Geschlechtsorgane | Erkrankungen der weiblichen Geschlechtsorgane | 0,03 % [5] |
| Atemwegserkrankungen | Atembeschwerden | 7,0 % [6] |
|  | Atemwegserkrankung | 5,0 % [12] |
|  | Atemstillstand | 4,0 % [9] |
| Schmerzen | Hals- und Brustschmerzen | 3,5 % [7] |
| Geplanter Krankenhausaufenthalt | Geplanter operativer Eingriff | 3,0 % [12] |
|  | Geplanter Krankenhausaufenthalt ohne operativen Eingriff | 2,0 % [12] |

1. Allers K, Hoffmann F (2018) Mortality and hospitalization at the end of life in newly admitted nursing home residents with and without dementia. Social Psychiatry and Psychiatric Epidemiology: The International Journal for Research in Social and Genetic Epidemiology and Mental Health Services 53:833-839

2. Bernardes C, Massano J, Freitas A (2018) Hospital admissions 2000–2014: A retrospective analysis of 288 096 events in patients with dementia. Archives of Gerontology & Geriatrics 77:150-157

3. Bickel H, Hendlmeier I, Hessler JB et al. (2018) The Prevalence of Dementia and Cognitive Impairment in Hospitals. Deutsches Arzteblatt international 115:733-740

4. Daiello LA, Gardner R, Epstein-Lubow G et al. (2014) Association of dementia with early rehospitalization among Medicare beneficiaries. Archives of gerontology and geriatrics 59:162-168

5. Fogg C, Griffiths P, Meredith P et al. (2018) Hospital outcomes of older people with cognitive impairment: An integrative review. International Journal of Geriatric Psychiatry 33:1177-1197

6. Givens JL, Selby K, Goldfeld KS et al. (2012) Hospital transfers of nursing home residents with advanced dementia. Journal of the American Geriatrics Society 60:905-909

7. Gungabissoon U, Perera G, Galwey NW et al. (2020) The association between dementia severity and hospitalisation profile in a newly assessed clinical cohort: the South London and Maudsley case register. BMJ open 10:e035779

8. Lin CS, Lin SY, Chou MY et al. (2017) Hospitalization and associated factors in people with Alzheimer's disease residing in a long-term care facility in southern Taiwan. Geriatrics & Gerontology International 17:50-56

9. Matsuoka T, Manabe T, Akatsu H et al. (2019) Factors influencing hospital admission among patients with autopsy‐confirmed dementia. Psychogeriatrics 19:255-263

10. Patira R, Zhao H, Azizi A (2018) A retrospective analysis of care in patients with dementia hospitalized at a tertiary medical center. Aging & mental health 22:773-777

11. Rudolph JL, Zanin NM, Jones RN et al. (2010) Hospitalization in community-dwelling persons with Alzheimer's disease: Frequency and causes. Journal of the American Geriatrics Society 58:1542-1548

12. Spears CC, Besharat A, Monari EH et al. (2019) Causes and outcomes of hospitalization in Lewy body dementia: A retrospective cohort study. Parkinsonism & Related Disorders 64:106-111

13. Zuliani G, Galvani M, Sioulis F et al. (2012) Discharge diagnosis and comorbidity profile in hospitalized older patients with dementia. Int J Geriatr Psychiatry 27:313-320

1. Ergebnisse aus Bickel et al. (2018) sind für Häufigkeiten nicht getrennt aufgeführt für MmD und MmCI [↑](#footnote-ref-1)
